# A challenging case of a pituitary macroadenoma and toxic thyroid adenoma with inappropriate TSH secretion

**DOI:** 10.1530/EDM-23-0136

**Published:** 2024-04-17

**Authors:** Michaela Despina Carides, Ruchika Mehta, Jaco Louw, Farzahna Mohamed

**Affiliations:** 1 Department of Internal Medicine, Faculty of Health Sciences, University of the Witwatersrand Johannesburg, Johannesburg, Gauteng, South Africa; 2 Faculty of Health Sciences, University of the Witwatersrand Johannesburg, Johannesburg, Gauteng, South Africa

**Keywords:** Geriatric, Female, Black - African, South Africa, Pituitary, Thyroid, Thyroid, Unique/unexpected symptoms or presentations of a disease, April, 2024

## Abstract

**Summary:**

Thyroid-stimulating hormone-secreting pituitary adenomas (TSHomas) are rare, accounting for less than 1% of all pituitary adenomas. We present a case of hyperthyroidism secondary to a likely TSHoma and coexisting functional thyroid adenoma. Laboratory errors and familial abnormalities in thyroid function tests were ruled out, and a diagnosis of the toxic thyroid adenoma was confirmed on a thyroid uptake scan. However, the triiodothyronine suppression test was contraindicated due to the patient’s cardiovascular disease, and the thyrotropin-releasing hormone stimulation test, measurement of glycoprotein hormone alpha-subunit, and genetic testing were unavailable. Magnetic resonance imaging of the brain revealed a suprasellar pituitary macroadenoma measuring 40 mm × 20.3 mm × 17 mm. The patient was initiated on carbimazole; however, thyroid stimulating hormone and thyroxine levels remained elevated. The patient declined trans-sphenoidal surgery and was treated with radioactive iodine to manage the toxic thyroid adenoma, leading to reduced thyroxine levels and symptom improvement. Unfortunately, the patient passed away before long-acting somatostatin analogs became available. This case highlights the diagnostic and therapeutic challenges involved in managing thyrotoxicosis with dual etiology.

**Learning points:**

- Hyperthyroidism can have multiple etiologies, which can coexist in the same patient.
- Persistent discordant thyroid function tests warrant further investigation.
- The gold standard for diagnosis of TSHomas remains immunohistochemical analysis of the tumor tissue.

## Background

Hyperthyroidism is typically caused by autoimmune thyroid disorders, toxic thyroid nodules, or goiters, but it can rarely be caused by a TSHoma, which autonomously secretes thyroid-stimulating hormone (TSH) (1). TSHomas are one of the least common causes of hyperthyroidism, representing 0.5–2% of all pituitary tumors, and are often misdiagnosed (1, 2). We highlight a rare case of a probable TSHoma coexisting with a toxic adenoma. Multiple pathologies can exist separately or have a causative link. Work-up and understanding of all etiologies of hyperthyroidism are critical in optimizing patient management. Diagnosis and treatment of TSHomas are outlined in the 2013 and updated 2019 European Guideline (2, 3).

## Case presentation

An 82-year-old woman presented to the endocrine department with hyperthyroidism of unknown etiology. She had a 1-month history of symptoms of congestive cardiac failure, as well as a 2-year history of a worsening frontal headache. It was not associated with a visual field defect. She reported vague symptoms of heat intolerance, but there was no history of anxiety, palpitations, insomnia, sweating, or diarrhea. She had a background history of hypertension for over 10 years, and her blood pressure was well controlled. She had a known diagnosis of ischemic heart disease for which she underwent coronary artery bypass graft surgery 9 years prior. She had been post-menopausal since the age of 52 and reported no family history of thyroid disease. On examination, she was normotensive with a tachycardia of 103 beats per minute. A small, symmetrical goiter without a bruit was observed, with no clinical features suggestive of Graves' disease. The patient was assessed to be in congestive cardiac failure, evidenced by pulmonary edema, elevated jugular venous pressure, and grade 2 bilateral pitting edema. An echocardiogram showed an ejection fraction of 50–55% with grade 3 diastolic dysfunction and pulmonary hypertension, with pulmonary artery pressures measuring 72 mm Hg.

## Investigations

Laboratory investigations (Table 1) showed an elevated thyroxine (FT4) level of 45.9 pmol/L (normal range (NR): 12.0–22.0; 3.57 ng/dL (NR: 0.93–1.71)) and triiodothyronine (FT3) level of 14.7 pmol/L (NR: 3.1–6.8; 0.96 ng/dL (NR: 0.2–0.44)), with an inappropriately elevated TSH level of 5.74 mIU/L (NR: 0.27–4.20). TSH receptor antibodies (Table 2), as well as antithyroid peroxidase (anti-TPO) and antithyroglobulin (anti-TG) antibodies, were negative. Potential laboratory errors, including assay or medication interference, were ruled out to the best of our capabilities as a cause for the discordant thyroid function tests.

**Table 1:** Thyroid function tests and peripheral markers of thyroid hormone pre- and post-RAI.

	Reference value	Pre-RAI	Post-RAI		
			2 months	5 months	6 months
TSH (mIU/L)	0.27–4.20	**5.74**	**13.24**	**22.17**	**38.51**
FT4 (pmol/L)	12–22	**45.9**	**38.3**	20	**24**
FT3 (pmol/L)	3.1–6.8	**14.7**	–	–	4
SHBG (nmol/L)	27.1–128.0	**131.6**	–	–	64.4
Total cholesterol (mmol/L)	<4.0	2.69	–	–	3.62
Ferritin (µg/L)	13–150	60	–	–	47
Bone specific ALP (µg/L)	7.0–22.4	**49.9**	–	–	–

Abnormal values are in bold.

**Table 2:** Laboratory results pre-RAI.

Parameters	Values	Normal range
ACTH (pmol/L)	4.2	1.6–13.9
08:00 AM cortisol (nmol/L)	392	133–537
IGF-1 (µg/L)	104.8	55.5–166.0
GH (µg/L)	1.2	0.13–9.88
E2 (pmol/L)	38	18–505
FSH (IU/L)	**<0.1**	16.7–113.6
LH (IU/L)	**<0.1**	16.7–113.6
PRL (µg/L)	**25.6**	4.8–23.3
Monomeric PRL (µg/L)	**19.6**	3.5–18.0
Thyroid antibodies		
TRAB, anti-TPO, anti-TG Ab	Negative	–
Short synacthen stimulation test		
Baseline cortisol (nmol/L)	284	–
Cortisol at 30 min (nmol/L)	480 (peak)	>403
Cortisol at 60 min (nmol/L)	375	–

Abnormal values are in bold.

Pituitary function tests (Table 2) were in keeping with central hypogonadism, with an estradiol of 38 pmol/L (NR: 18–505; 10.34 pg/mL (NR: 5–138)), progesterone 0.2 nmol/L (NR: 0.1–0.4; 0.06 ng/mL (NR: 0.05–0.13)), and an inappropriately low follicle-stimulating hormone (FSH) of less than 0.10 IU/L (NR: 25.8–134.8 IU/L) and luteinizing hormone (LH) of less than 0.10 IU/L (NR: 7.7–58.5 IU/L). Morning (08:00 AM) serum cortisol was indeterminate at 392 nmol/L (NR: 133–537 nmol/L; 14.21 μg/dL (NR: 4.82–19.47)), and serum adrenocorticotropic hormone (ACTH) was normal at 4.2 pmol/L (NR: 1.6–13.9; 19.09 pg/mL (NR: 7.2 –63.3)) (4, 5). The short synacthen stimulation test excluded secondary adrenal insufficiency with a peak cortisol of 480 nmol/L (cutoff 441; 17.4 µg/dL cutoff 15.99) at 30 min (4, 5, 6). Prolactin was mildly increased at 25.6 µg/L (NR: 4.8–23.3; 1113.04 pmol/L (NR: 208.69–1013.04)), and insulin-like growth factor 1 (IGF-1) levels were normal at 104.8 ng/mL (NR: 33.6–177.8; 13.6 nmol/L (NR: 4.37–23.11)).

Peripheral markers of thyroid hormone (Table 1) showed a marginally elevated sex hormone-binding globulin (SHBG) at 131.6 nmol/L (NR: 27.1–128.0; 12.50 μg/mL (NR: 2.57–12.16)) and a bone-specific alkaline phosphatase (bsALP) of more than twice the upper limit of normal at 49.9 μg/L (NR: 7.0–22.4). Total cholesterol was 2.69 mmol/L (NR: <4.0; 104.00 mg/dL (NR: <154.64)), triglycerides were 0.68 mmol/L (NR: <1.7; 60.18 mg/dL (NR: <150.45)), and ferritin levels were 60 μg/L (NR: 13–150; 134.83 pmol/L (NR: 29.21–337.08)), all within normal ranges.

**Figure 1:**
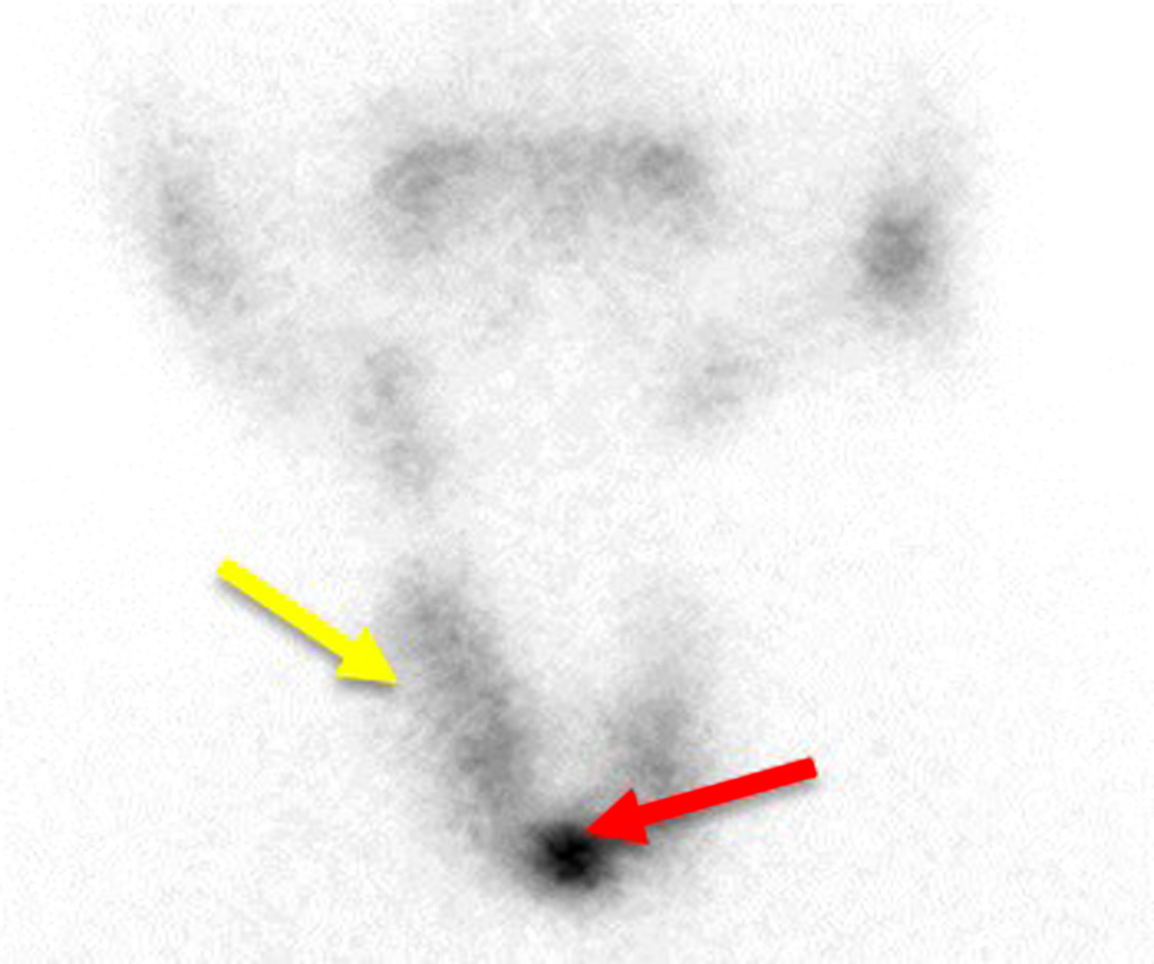
Thyroid uptake scan on admission showing a solitary hot nodule (red arrow) overlying the isthmus with a background increased uptake (yellow arrow).

**Figure 2:**
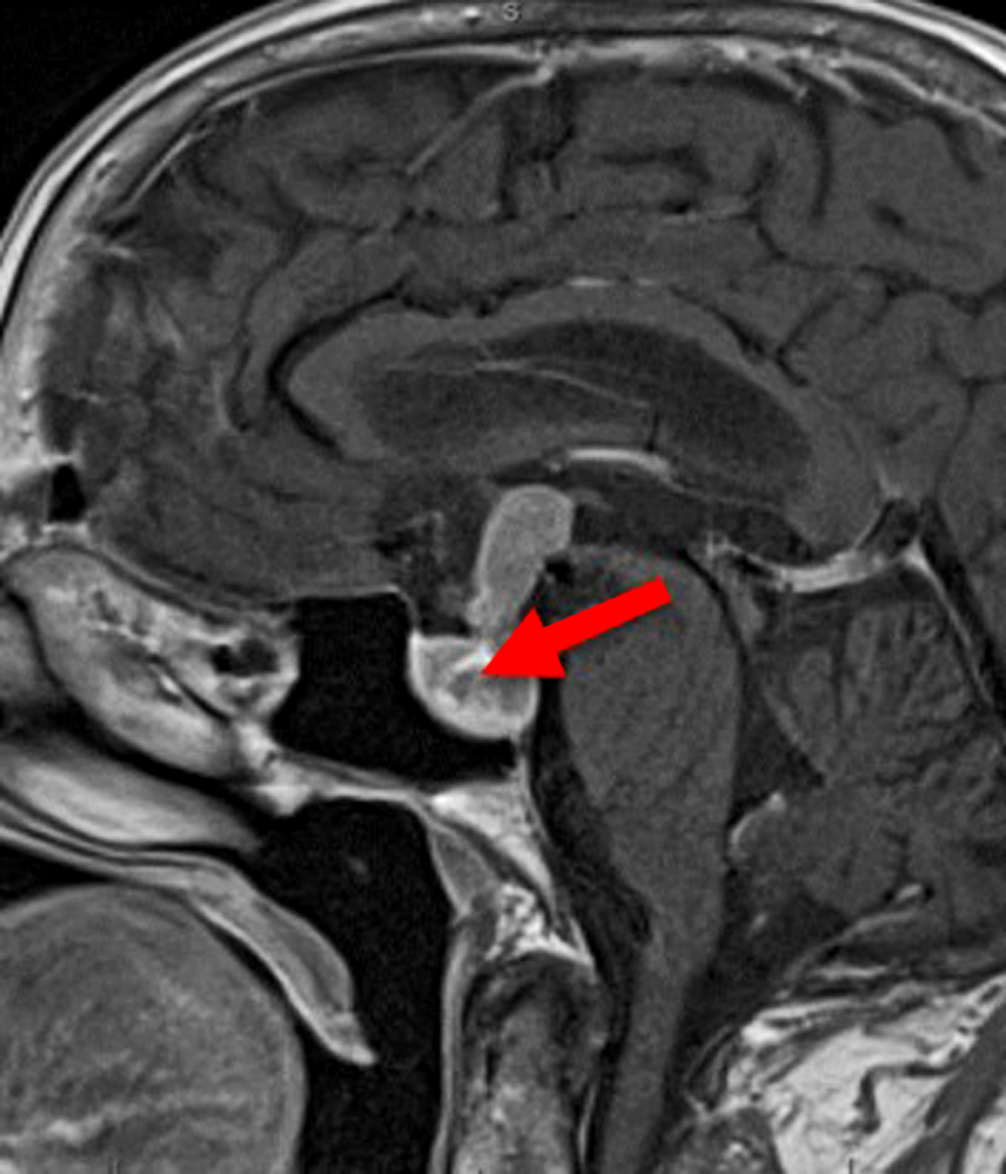
Sagittal view of the pituitary gland on a brain MRI. Arrow shows a 40 × 20.3 × 17 mm suprasellar pituitary macroadenoma associated with mild obstructive hydrocephalus.

Radiological imaging included a technetium thyroid uptake scan, which showed a multinodular goiter with a prominent hot nodule in the region of the isthmus and the presence of a diffusely non-suppressed thyroid background (Fig. 1). Ultrasound-guided fine-needle aspiration of this hot nodule confirmed a benign follicular nodule. Magnetic resonance imaging (MRI) of the brain revealed a 40 mm × 20.3 mm × 17 mm suprasellar pituitary macroadenoma associated with mild obstructive hydrocephalus postulated to be a TSHoma (Fig. 2). Based on these findings, along with elevated peripheral markers of thyroid hormone, the diagnosis of a coexisting TSHoma was favored over resistance to thyroid hormone (RTH).

### Treatment

The patient was initiated on a diuretic and carbimazole. At the time, long-acting somatostatin analogs (SSAs) were not available. Optimal work-up and management were decided upon by a multidisciplinary team, considering that the patient had declined a trans-sphenoidal surgery (TSS) of the pituitary. Despite titrating up carbimazole, the patient’s FT4 levels remained high, and the decision was made to manage the coexisting pathology of the toxic thyroid adenoma. She underwent radioactive iodine ablation (RAI) to provide some degree of reduction in thyroid hormone levels.

### Outcome and follow-up

**Figure 3:**
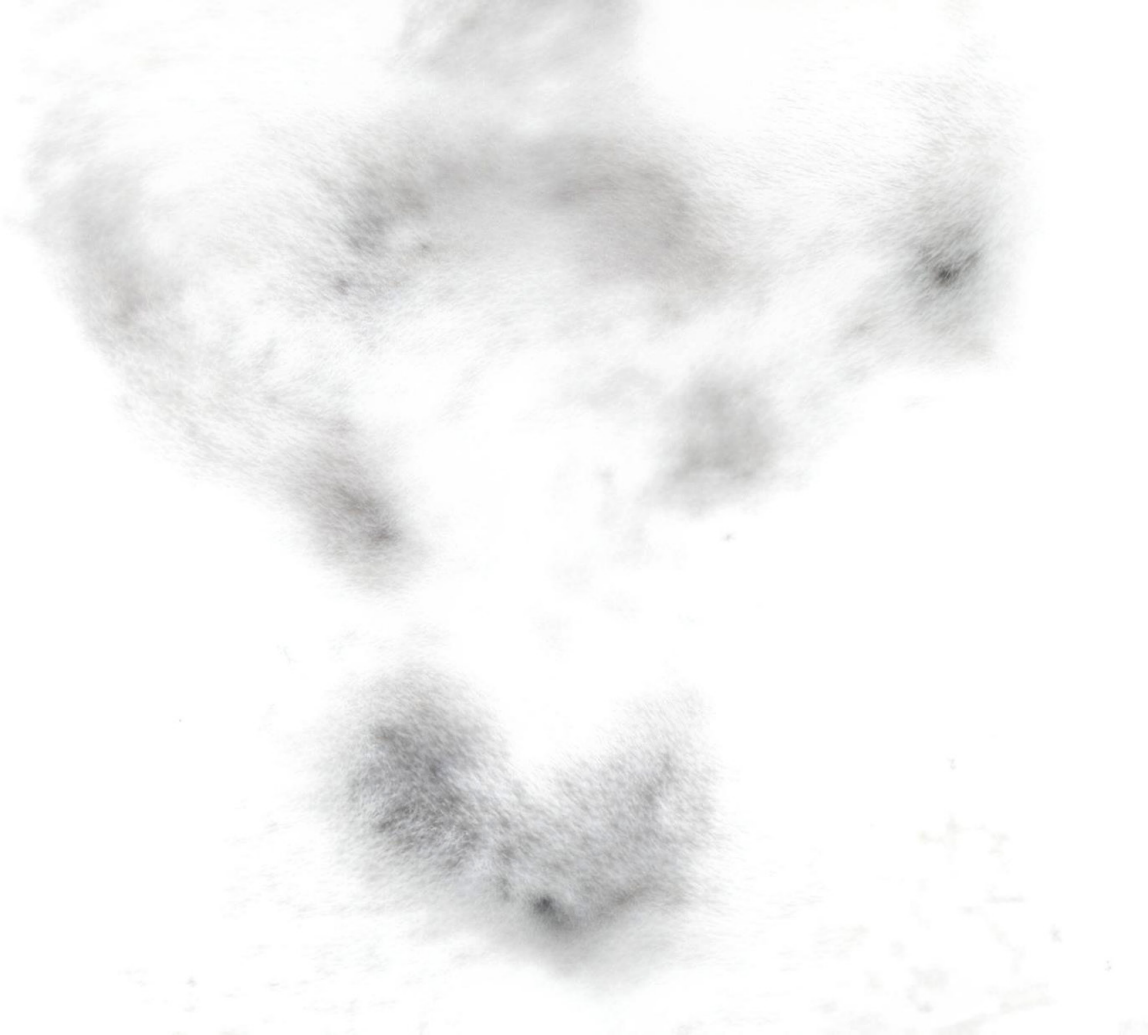
Thyroid uptake scan 6 months post radioactive iodine ablation showing inhomogeneous uptake of the tracer, with the resolution of the solitary hot nodule.

Thyroid function tests at 5 months post-RAI (Table 1) showed a normal FT4 of 20 pmol/L (NR: 12.0–22.0; 1.55 ng/dL (NR: 0.93–1.71)) and an elevated TSH of 22.17 mIU/L (NR: 0.27–4.20). At 6 months post RAI, the TSH was markedly increased with increasing FT4 levels. Repeat thyroid uptake scan showed both lobes of the thyroid gland having slightly inhomogeneous uptake of the tracer, and the previous hot nodule in the isthmus had resolved (Fig. 3) (7). An octreotide suppression test and Ga-68DOTATATE positron emission tomography (PET)/CT were scheduled while awaiting the availability of the long-acting SSA. Unfortunately, the patient passed away prior to this.

## Discussion

In 1960, the link between the pituitary and excessive TSH production causing secondary hyperthyroidism was proposed (7). However, it was not until 1980 that a few case reports confirmed TSHoma (7). Central hyperthyroidism is defined as uninhibited TSH secretion on a background of elevated thyroid hormone levels (3, 8). The main differentials for this abnormal feedback in the hypothalamus–pituitary–thyroid axis are autonomous TSH secretion from a TSHoma or resistance to thyroid hormone (RTH) (3, 7).

The first step in the work-up of central hyperthyroidism is to exclude assay interference. According to Hattori *et al.,* macro TSH is a large molecular-sized TSH that is mostly a complex of TSH and IgG (9). Macro TSH is interpreted by routine TSH immunoassays as an elevated serum TSH level, commonly referred to as assay interference (9). This is observed in all commercial TSH assay platforms (9). To exclude suspected macro TSH, we performed a polyethylene glycol precipitation on a Roche COBAS 8000 602 instrument. The method is a heterogeneous electrochemiluminescent immunoassay employing monoclonal mouse antibodies. Heterophile antibodies were excluded by running the sample first on the Roche COBAS and then on a Siemens Centaur, which employs both mouse (monoclonal) and sheep (polyclonal) antibodies. The two results displayed a difference of less than total allowable error (TAE), indicating that the minor difference is simply due to analytical variation. At the time of testing, TAE was 12.4%, as per the European Federation of Clinical Chemistry and Laboratory Medicine’s biological variation database (10).

Distinguishing between a TSHoma and RTH can be challenging, but dynamic tests (i.e. the T3 suppression test or the thyrotropin releasing hormone (TRH) stimulation test) or genetic testing for the TRβ gene can aid in the diagnosis (3, 8). The T3 suppression test is used for diagnosis and excluding non-functioning pituitary adenomas, but it is not necessary if a macroadenoma is found on MRI along with high FT4, normal TSH, and abnormal TRH stimulation test (7). These patients should be referred directly for surgery (7). However, the test should be avoided in patients with severe pulmonary hypertension, cardiovascular disease, or any other conditions that may decompensate (7). Other factors such as family history, peripheral tissue metabolic markers, and imaging findings can aid in the diagnosis.

Peripheral tissue metabolic markers for hyperthyroidism have previously been used to distinguish TSHomas from RTH (11). However, since this case was complicated by a toxic thyroid adenoma, the diagnostic value of these markers was limited. Patients with TSHomas typically present with elevated SHBG, bs-ALP, carboxy-terminal cross-linked telopeptide of type I collagen (ICTP), and ferritin. Cholesterol levels in patients presenting with TSHomas are typically normal or low (11).

MRI of the pituitary is only recommended if dynamic testing is suggestive of a TSHoma (1). The majority of TSHomas are macroadenomas invading surrounding structures, and a review of 535 cases of TSHomas revealed a mean diameter of 21.5 ± 7.9 mm in 76% of cases (1). The presence of a macroadenoma, especially with elevated serum glycoprotein hormone alpha-subunit (α-GSU), strongly indicates a TSHoma, while there is no correlation between adenoma diameter and FT4 or TSH levels (1, 3). However, 20% of patients with RTH may have an incidental finding of a pituitary lesion on MRI (7). As with our patient, suprasellar extension and thus signs and symptoms of an expanding tumor mass are predominant in many patients. Headache is reported in 20–25% of patients, and visual field defects in about 50% of patients presenting with a TSHoma (3). In the reported case, the probable diagnosis of a TSHoma as opposed to RTH was made based on multiple factors such as the absence of RTH on family history, serum peripheral tissue metabolic markers for hyperthyroidism, and imaging consistent with a pituitary fossa macroadenoma. Although not mandatory, scintigraphy with radio-labeled octreotide is commonly used for functional imaging in TSHomas due to their high expression of somatostatin receptors (2).

Mixed adenomas, characterized by hypersecretion of other anterior pituitary hormones, are observed in 20–25% of TSHomas, leading to complications like central hypogonadism (3). The central hypogonadism in these patients can be due to autonomous secretion of prolactin or mass effect from the tumor resulting in elevated prolactin levels from stalk effect, which was the likely cause of elevated prolactin in our patient (3).

Uninhibited stimulation of the thyroid gland by TSH may rarely contribute to the development of an adenoma as described by Aksoy *et al.* in a case report of an incidental TSHoma and coexisting functional thyroid adenoma (12). It is noted that TSHomas may increase the risk of thyroid cancer, highlighting the importance of excluding neoplastic lesions through ultrasound and fine needle aspiration (7). However, the majority of hyperfunctioning nodules are benign, with the occurrence rate of a follicular carcinoma in a hot nodule being approximately 1% (13).

Trans-sphenoidal pituitary resection is the primary treatment for TSHomas (7). Radiotherapy as an adjunct to surgery shows some benefit (7). The best medical treatment includes long-acting SSAs, such as octreotide, which results in the restoration of a euthyroid state in more than 90% of patients (3).

The decision to offer RAI in our patient was based on the patient’s refusal of TSS, the lack of access to long-acting SSAs, and the presence of the toxic adenoma with uncontrolled FT4 levels despite carbimazole titration. However, it is important to note that inappropriate thyroid ablation can promote the development of a macroadenoma, similar to the mechanism of Nelson syndrome in ACTH-producing tumors when cortisol feedback is removed (7). Limited data are available on the use of RAI for a TSHomas. However, Daousi *et al.* have reported the successful RAI treatment of two patients known with TSHomas with no marked changes in tumor size for 12 years of follow-up (14).

Diagnosing TSHomas is challenging, but advances in assay methods, imaging, and long-acting SSAs have significantly improved the diagnosis and treatment of these rare adenomas (7). TSS is the gold standard for diagnosis and treatment; however, patient preference and willingness to undergo surgery are always of utmost importance.

## Declaration of interest

The authors declare that there is no conflict of interest that could be perceived as prejudicing the impartiality of this case report.

## Funding

This work did not receive any specific grant from any funding agency in the public, commercial, or non-for-profit sector.

## Patient consent

Written informed consent for the publication of clinical details and clinical images was obtained from the patient.

## Author contribution statement

All authors made an individual and equal contribution to the authorship of this case report. Furthermore, all authors reviewed and approved the final draft.
